# Integrated Advanced Monitoring and Target-Controlled Infusion Anesthesia in a Child With Arthrogryposis Multiplex Congenita

**DOI:** 10.7759/cureus.109565

**Published:** 2026-05-24

**Authors:** Sivalakshmi Ramesh, Kishore Manivannan, Aruna Parameswari, Mahesh Vakamudi

**Affiliations:** 1 Anesthesiology, Sri Ramachandra Institute of Higher Education and Research, Chennai, IND

**Keywords:** arthrogryposis multiplex congenita, difficult airway, neuromuscular monitoring, pediatric anesthesia, sugammadex, target-controlled infusion

## Abstract

Arthrogryposis multiplex congenita (AMC) is associated with significant anesthetic challenges due to multisystem involvement, craniofacial abnormalities leading to potential difficult airway, thermoregulatory instability, and concerns regarding susceptibility to malignant hyperthermia. Current perioperative practice emphasizes advanced airway planning, quantitative neuromuscular monitoring, tailored hypnotic delivery, and reliable neuromuscular reversal. We describe the perioperative management of a child with AMC using an integrated, technology-guided anesthetic strategy. A 10-year-old, 33.5 kg male with AMC and retrognathia presented for corrective lower-limb orthopedic surgery. He had a previous history of intraoperative hyperthermia. Standard American Society of Anesthesiologists (ASA) monitoring was augmented with entropy-guided depth of anesthesia, quantitative train-of-four (TOF) monitoring, and invasive arterial pressure monitoring. Anesthesia was induced and maintained using target-controlled infusion of propofol and remifentanil. Tracheal intubation was successfully achieved using videolaryngoscopy following rocuronium administration. Multimodal analgesia included a single-shot caudal block with bupivacaine and clonidine. Entropy values were maintained between 40 and 60, with stable hemodynamics and normothermia throughout the procedure. At the end of surgery, sugammadex was administered at a TOF ratio of 0.4, achieving complete neuromuscular recovery prior to awake extubation. Postoperative recovery was uneventful. This case highlights the role of advanced monitoring, sugammadex-facilitated reversal, videolaryngoscopy, and regional anesthesia in enhancing perioperative safety in pediatric patients with AMC.

## Introduction

Arthrogryposis multiplex congenita (AMC) is a heterogeneous, non-progressive disorder characterized by multiple joint contractures and muscle involvement; craniofacial and maxillofacial structures may be affected in up to a quarter of patients, with reduced mouth opening and mandibular hypomobility [1]. Airway difficulty in AMC can be profound, including cannot ventilate-cannot intubate scenarios necessitating early transition to surgical airway in emergencies [2]. Perioperative management is further complicated by difficult venous access and positioning, potential cardiorespiratory comorbidities, and concerns regarding hyperthermia and possible malignant hyperthermia (MH) susceptibility, though the association remains debated [3,4].

Current guidance underscores the centrality of quantitative neuromuscular monitoring to titrate block depth and document recovery (train-of-four ratio ≥0.9) and highlights the advantages and limitations of neostigmine and sugammadex for antagonism [5]. In pediatric patients, a systematic review suggests sugammadex provides rapid and effective reversal of rocuronium-induced block without increased adverse events compared with neostigmine [6]. Propofol's pharmacokinetic profile supports the use of total intravenous anesthesia (TIVA)/TCI techniques and allows reliable clinical titration [7]. Regional analgesia complements anesthesia in AMC and may improve postoperative outcomes [8].

We report a pediatric AMC case managed with an integrated strategy: entropy-guided TCI propofol-remifentanil, quantitative neuromuscular monitoring with rocuronium and sugammadex reversal, video laryngoscopy for airway management, invasive arterial pressure monitoring, and caudal analgesia. Informed consent to publication was obtained from the patient's parents.

## Case presentation

A 10-year-old boy weighing 33.5 kg with arthrogryposis multiplex congenita presented for elective corrective lower-limb orthopedic surgery. Since birth, he had experienced restricted limb movements and difficulty walking. Antenatal history was significant for oligohydramnios, and he was delivered preterm at 32 weeks of gestation, requiring neonatal intensive care unit admission for 15 days. During the previous surgery in 2017, he underwent bilateral knee soft-tissue contracture release, and the patient developed intraoperative hyperthermia with a maximum recorded temperature of 38.8 degrees Celsius. There was no associated hypercarbia, muscle rigidity, arrhythmia, cola-coloured urine, or unexplained metabolic acidosis. The episode responded to conservative measures, including active cooling and intravenous fluids, and dantrolene was not required. There was no history of recurrent pneumonia, seizures, cyanosis, drug allergy, or recent upper respiratory tract infection.

Preoperative examination revealed bilateral popliteal contractures and valgus deformity of the left elbow. Airway assessment showed retrognathia, Mallampati grade 2, adequate mouth opening, normal neck mobility, and normal dentition without loose teeth or protruding teeth. Systemic examination was otherwise unremarkable. Baseline vital parameters included a heart rate of 101/min, blood pressure of 100/60 mmHg, respiratory rate of 18/min, and peripheral oxygen saturation (SpO₂) of 99% on room air. Routine hematological and biochemical investigations were within normal limits. High-risk informed consent was obtained from the parents after discussing the possibility of difficult airway management and other perioperative complications. Fasting guidelines were followed according to institutional protocol, with six hours for solids and two hours for clear fluids. In the operating room, standard American Society of Anesthesiologists monitors were applied, and invasive arterial blood pressure monitoring was established via left radial artery cannulation. Anesthesia was induced using target-controlled infusion (TCI) of propofol (effect-site concentration 3.5 ug/mL) and remifentanil 3 ng/mL. Remifentanil was selected because of its rapid onset, short context-sensitive life, and ease of titration during TCI-based anesthesia, allowing precise hemodynamic control and rapid recovery. Baseline entropy values before induction were 96-98, and entropy electrodes were placed over the forehead according to manufacturer recommendations. Neuromuscular blockade was achieved with rocuronium 0.6 mg/kg under quantitative train-of-four (TOF) monitoring at the adductor pollicis muscle. Tracheal intubation was successfully achieved on the first attempt using videolaryngoscopy (Cormack Lehane grade -1 view) with a 5.5 mm cuffed flexometallic endotracheal tube without airway trauma. Anesthesia was maintained with oxygen and air (2:1) along with continued TCI propofol and remifentanil infusion, titrated to maintain entropy values between 40 and 60. A single-shot caudal epidural block was administered using 0.25% bupivacaine 1 mL/kg with clonidine 15 µg for intraoperative and postoperative analgesia. Intraoperative hemodynamic parameters and temperature remained stable throughout the procedure, with no evidence of arrhythmia or hypercarbia. End tidal carbon dioxide values were maintained between 32-38 mmHg throughout the procedure. Positioning was achieved without difficulty, and no pressure-related complications were observed. At the conclusion of surgery, the TOF ratio was 0.4, and neuromuscular blockade was reversed with sugammadex 2 mg/kg. Following recovery of the TOF ratio to ≥0.9, the trachea was extubated after confirming adequate spontaneous respiration and intact airway reflexes. The patient was transferred to the post-anesthesia care unit for further observation. He remained hemodynamically stable with satisfactory analgesia and was subsequently shifted to the ward. The postoperative course was uneventful. The patient's preoperative clinical characteristics and airway assessment findings are summarized in Table 1.

**Table 1:** Preoperative airway assessment

Preoperative assessment	Findings
Age/weight	10 years / 33.5 kg
Diagnosis	Arthrogryposis multiplex congenita (AMC)
Surgery	Elective corrective lower-limb orthopedic surgery
Airway	Retrognathia; adequate mouth opening, normal dentition, normal neck mobility, Mallampati grade-2
Relevant history	Intraoperative hyperthermia (2017, resolved conservatively); preterm birth at 32 weeks
Baseline vitals	HR 101/min, BP 100/60 mmHg, SpO₂ 99% RA, RR 18/min

The intraoperative anesthetic management and monitoring parameters are summarized in Table 2

**Table 2:** Intraoperative monitoring parameters TCI - target-controlled infusion; TOF - train-of-four

Intraoperative monitoring	Values
Anesthetic technique	TCI propofol 3.5 µg/ml (effect-site) + remifentanil 3 ng/ml; entropy 40–60
Neuromuscular block	Rocuronium 0.6 mg/kg; quantitative TOF at adductor pollicis; TOF ratio 0.4 at reversal
Reversal	Sugammadex 2 mg/kg; TOF ratio ≥0.9 achieved before extubation
Regional analgesia	Caudal block: bupivacaine 0.25% 1 ml/kg + clonidine 15 μg
Temperature	Stable throughout; no hyperthermia
Hemodynamics	Stable; no arrhythmia or hypercarbia

Differential diagnosis

In a child with AMC presenting with retrognathia and a prior history of intraoperative hyperthermia, the differential considerations for anesthetic planning included: 1) difficult or failed airway secondary to craniofacial anomaly; 2) malignant hyperthermia (MH) susceptibility versus non-specific intraoperative hyperthermia; and 3) residual neuromuscular blockade contributing to respiratory compromise at extubation. Clinical evaluation supported retrognathia as the primary airway predictor; MH susceptibility was considered unlikely based on available evidence but could not be excluded, prompting avoidance of triggering agents; and quantitative TOF monitoring with sugammadex reversal addressed the risk of residual block.

## Discussion

Arthrogryposis multiplex congenita (AMC) presents several anesthetic challenges, particularly difficult airway management, altered musculoskeletal anatomy, positioning difficulties, and concerns regarding thermoregulatory instability. Craniofacial abnormalities such as retrognathia and restricted mandibular mobility may contribute to difficult mask ventilation and intubation [1,2]. In our patient, videolaryngoscopy was incorporated into the primary airway plan because of retrognathia and anticipated airway difficulty, enabling successful first-attempt intubation. A previous episode of intraoperative hyperthermia prompted careful perioperative temperature monitoring and avoidance of potential triggering agents. Although an association between AMC and malignant hypertheria (MH) remains controversial, vigilance is recommended because episodes of perioperative hyperthermia have been described in these patients [3,4]. In our case, temperature and end-tidal carbon dioxide remained within normal limits throughout surgery, and no clinical features suggestive of MH were observed. Quantitative neuromuscular monitoring is essential in patients receiving non-depolarising neuromuscular blockers because clinical assessment alone may fail to detect residual blockade [5]. Rocuronium was administered under quantitative train-of-four (TOF) monitoring, and sugammadex was used for reversal at a TOF ratio of 0.4. Recovery to a TOF ratio ≥0.9 was confirmed before extubation, minimizing the risk of postoperative respiratory complications. Previous pediatric studies have demonstrated that sugammadex provides rapid and reliable reversal of rocuronium-induced neuromuscular blockade with shorter recovery times compared with neostigmine [6]. Propofol has high lipid solubility, a large volume of distribution, physiologically based pharmacokinetic modeling, potential pulmonary sequestration during bolus dosing, and the impact of body composition on distribution and recovery [7]. Target-controlled infusion (TCI) using propofol and remifentanil anesthesia allowed controlled titration of hypnosis and analgesia while avoiding volatile anesthetic agents. Entropy monitoring facilitated maintenance of an adequate depth of anesthesia throughout the procedure. Remifentanil was particularly useful because of its rapid onset, easy titratability, and short context-sensitive half-life, allowing hemodynamic stability and rapid postoperative recovery. Remifentanil complements TCI propofol by providing titratable intraoperative analgesia with rapid offset, which is advantageous in pediatric patients undergoing prolonged orthopedic procedures. Regional analgesia forms an important component of multimodal perioperative care in AMC [8]. The caudal epidural block with bupivacaine and clonidine provided effective intraoperative and postoperative analgesia while reducing perioperative opioid requirements. The combined use of videolaryngoscopy, entropy-guided TCI anesthesia, quantitative neuromuscular monitoring, sugammadex reversal, invasive arterial monitoring, and regional analgesia contributed to an uneventful perioperative course in this child with AMC.

## Conclusions

Arthrogryposis multiplex congenita poses multiple concurrent anesthetic challenges, including difficult airway, thermoregulatory instability, and complex analgesic requirements. Video laryngoscopy should be incorporated as the primary airway plan when craniofacial predictors of difficult laryngoscopy are present. Quantitative neuromuscular monitoring is essential to titrate block depth and document recovery to a TOF ratio ≥0.9 before extubation. Sugammadex provides rapid and reliable reversal of rocuronium-induced neuromuscular block in children and should be guided by quantitative monitoring. A multimodal strategy combining entropy-guided TCI propofol-remifentanil, quantitative neuromuscular monitoring, and regional analgesia can achieve safe perioperative management in pediatric AMC.
